# A Preliminary Study of the Efficacy of Transcranial Direct Current Stimulation in Trigeminal Neuralgia

**DOI:** 10.3389/fnhum.2022.848347

**Published:** 2022-03-04

**Authors:** Babak Babakhani, Narges Hoseini Tabatabaei, Kost Elisevich, Narges Sadeghbeigi, Mojtaba Barzegar, Neda Mohammadi Mobarakeh, Fatemeh Eyvazi, Zahra Khazaeipour, Arman Taheri, Mohammad-Reza Nazem-Zadeh

**Affiliations:** ^1^Brain and Spinal Cord Injury Research Centre, Neuroscience Institute, Tehran University of Medical Sciences, Tehran, Iran; ^2^Medical School, Tehran University of Medical Sciences, Tehran, Iran; ^3^Department of Clinical Neurosciences, Spectrum Health, College of Human Medicine, Michigan State University, Grand Rapids, MI, United States; ^4^National Brain Mapping Laboratory, Tehran, Iran; ^5^Intelligent Quantitative Biomedical Imaging L.L.C, Tehran, Iran; ^6^Medical Physics and Biomedical Engineering Department, Tehran University of Medical Sciences, Tehran, Iran; ^7^Research Center for Molecular and Cellular Imaging, Advanced Medical Technologies and Equipment Institute, Tehran University of Medical Sciences, Tehran, Iran; ^8^Cognitive Psychology Department, Shahid Beheshti University, Tehran, Iran

**Keywords:** treatment efficacy, fMRI, neural bases, tDCS—transcranial direct current stimulation, brain stimulation, dMRI (diffusion magnetic resonance imaging), pain, trigeminal neuralgia

## Abstract

The purpose of this study is to assess the efficacy of transcranial direct current stimulation (tDCS) in patients with treatment-refractory trigeminal neuralgia (TN) and examine the utility of neuroimaging methods in identifying markers of such efficacy. Six patients with classical TN refractory to maximal medical treatment, underwent tDCS (three cases inhibitory/cathodic and three cases excitatory/anodic stimulation). All patients underwent pre- and posttreatment functional magnetic resonance imaging (fMRI) during block-design tasks (i.e., Pain, Pain + tDCS, tDCS) as well as single-shell diffusion MRI (dMRI) acquisition. The precise locations of tDCS electrodes were identified by neuronavigation. Five therapeutic tDCS sessions were carried out for each patient with either anodic or cathodic applications. The Numeric Rating Scale of pain (NRS) and the Headache Disability Index (HDI) were used to score the subjective efficacy of treatment. Altered activity of regional sites was identified by fMRI and associated changes in the spinothalamocortical sensory tract (STCT) were measured by the dMRI indices of fractional anisotropy (FA) and mean diffusivity (MD). Fiber counts of the bilateral trigeminal root entry zone (REZ) were performed as an added measure of fiber loss or recovery. All patients experienced a significant reduction in pain scores with a substantial decline in HDI (*P* value < 0.01). Following a course of anodic tDCS, the ipsilateral caudate, globus pallidus, somatosensory cortex, and the contralateral globus pallidus showed a significantly attenuated activation whereas cathodic tDCS treatment resulted in attenuation of the thalamus and globus pallidus bilaterally, and the somatosensory cortex and anterior cingulate gyrus contralaterally. dMRI analysis identified a substantial increase (>50%) in the number of contralateral sensory fibers in the STCT with either anodic or cathodic tDCS treatment in four of the six patients. A significant reduction in FA (>40%) was observed in the ipsilateral REZ in the posttreatment phase in five of the six patients. Preliminary evidence suggests that navigated tDCS presents a promising method for alleviating the pain of TN. Different patterns of activation manifested by anodic and cathodic stimulation require further elaboration to understand their implication. Activation and attenuation of responses at various sites may provide further avenues for condition treatment.

## Introduction

Trigeminal neuralgia (TN) is a chronic neuralgic facial pain disorder that involves the territory of one or more branches of the trigeminal nerve and affects seniors and women more than men in a ratio of 3:1 with a prevalence of 0.03–0.3% (Cheshire, 2007; Olesen et al., 2013; Sivakanthan et al., 2014; Cheshire<suffix>Jr.</suffix>, 2015; De Toledo et al., 2016). Added to the pain of the condition, patients with TN have an increased risk of anxiety and depression with significant life consequences (Morra et al., 2016). A variety of pathologies underlie the occurrence of TN with genetic, biologic and environmental factors implicated in its evolution affecting changes within both the central nervous system and nerve root itself (Pollack et al., 1988; Duff et al., 1999; Fleetwood et al., 2001; Devor et al., 2002; Gupta et al., 2002; Smyth et al., 2003; Hall et al., 2006; Hemminki et al., 2007; El et al., 2008; Ebner et al., 2010; Zakrzewska and Linskey, 2014).

Medical treatment is considered as firstline therapy for TN with preferences given to antiepileptic drugs and baclofen. Side effects of treatment with such drugs are typically dose-related (Zakrzewska, 2001) and age-related complications, intolerance of medical therapy, progression of pain severity, and the relapsing nature of TN limit efficacy. Evidence has identified neuromodulation as a potentially effective treatment of such conditions (Hansen et al., 2011; Hagenacker et al., 2014).

The processing of pain stimuli involves a complex arrangement of sites within the cerebral hemispheres that are accessible to neuromodulation. Reciprocal connections exist both between motor (MC) and premotor (PM) cortices and between the PM and ventrolateral nucleus (VLN) of the thalamus (Bosch-Bouju et al., 2013). Both the PM and globus pallidus interna (GPi) project to the anterior VLN while both the MC and cerebellum project to the posterior VLN. The ventromedial nucleus (VMN) of the thalamus similarly receives input from the PM as well as the substantia nigra pars reticulata (SNpr). Striatal GABAergic spiny neurons exert an inhibitory effect upon nigrothalamic and pallidothalamic neurons (Ueki et al., 1977; Uno et al., 1978; Chevalier and Deniau, 1982; Yamamoto et al., 1983) resulting in a disinhibition of the VMN and its projection onto the MC. The ventroposterior nucleus pars medialis (VPM) of the thalamus similarly has reciprocal connections with the somatosensory cortex (SSC) (Sherman and Guillery, 2011). It receives sensory information from the principal trigeminal nucleus. The posteromedial nucleus (POm) receives sensory information from the spinal trigeminal nucleus and from the SSC (Veinante et al., 2000; Sherman and Guillery, 2011; Groh et al., 2014). The POm additionally regulates SSC processing between it and secondary sensory cortical areas (Liao and Yen, 2008; Yam et al., 2018).

The basal ganglia-thalamus-cerebral cortex circuit consists of fibers projecting from the supplementary motor area (SMA), PM, MC, and SSC to the putamen which projects to both external and internal segments of the globus pallidus The circuit is completed with efferents from the GPi to the VLN and back to the SMA (Jürgens, 1984; Alexander et al., 1986). Two distinct pathways, direct and indirect, from the basal ganglia regulate the thalamic response with opposing effect. Activation of the striatum inhibits GPi neurons causing the direct pathway to release thalamic neurons from inhibition, and to excite the MC. The indirect pathway involves striatal inhibition of the external globus pallidus (GPe) which then disinhibits neurons of the subthalamic nucleus to bring about excitation of the GPi. The latter results in an inhibition of the thalamus and subsequent inhibition of the MC.

Medical treatment is considered as firstline therapy for TN with preferences given to antiepileptic drugs (AEDs) and baclofen. Side effects of treatment with such drugs are typically dose-related and underestimated by clinicians (Zakrzewska, 2001). Age-related complications, intolerance of medical therapy, progression of pain severity and the relapsing nature of TN limit efficacy. Nonmedical treatment has primarily involved minimally invasive surgical procedures while neuromodulatory methods have more recently become acknowledged as effective interventions (Hansen et al., 2011; Hagenacker et al., 2014). Stimulation of the MC (Attal et al., 2016) and caudate nucleus (Ervin et al., 1966; Lineberry and Vierck, 1975) have both been shown to mitigate pain. Noxious stimulation activates the SSC bilaterally but does so more ipsilaterally (Becerra et al., 2008).

A recent review analyzed the therapeutic effect of repeated transcranial magnetic stimulation (rTMS) and transcranial direct current stimulation (tDCS) on different types of chronic headache (Stilling et al., 2019). Cases of mild-moderate grade headache have responded with a reduced frequency of events using anodic tDCS applied to either the left MC or the dorsolateral prefrontal cortex (DLPFC) with the cathode overlying the contralateral fp2 site of the 10-20 EEG system. Orofacial pain disorders likewise have responded to similar stimulation of the MC, DLPFC, and the secondary sensory cortex (Ferreira et al., 2019).

Different applications of navigated tDCS in six patients with treatment-refractory TN, specifically the locations of anode and cathode, were evaluated to determine response to treatment. Magnetic resonance imaging was undertaken to identify coincident structural and functional changes underlying the effect of the stimulation. Activation and attenuation of responses at various sites and with a larger TN cohort may provide further avenues for treatment of the condition.

## Materials and Methods

### Study Design

This research study was approved by Institutional Review Board (IRB) of the Tehran University of Medical Sciences. Six otherwise healthy patients with unilateral primary TN (five left, one right; Table S1) refractory to maximal medical therapy (including Carbamazepine, Pregabalin, and Clonidine) were enrolled in the study following informed consent. None suffered from depression or other psychological disorder. All had failed to achieve treatment goals with recommended standard medical treatment (International Headache Society). Inclusion eligibility was drawn from the criteria established by the Headache Classification Committee for TN [International Headache Society (HIS)] (Ettlin, 2013); specifically, recurrent paroxysmal unilateral facial pain in the distribution(s) of one or more divisions of the 5th cranial nerve, without radiation beyond. Attacks of neuralgiform pain can be precipitated by innocuous stimuli within the affected territory of trigeminal division. Morphological changes of Trigeminal nerve are evident in MRI study. Exclusion criteria included: (1) Presence of any severe systemic comorbidity, (2) History of arrhythmia or seizure, and (3) Any contraindication for magnetic resonance imaging (MRI). Both SSC and MC were determined using standard neuronavigation technique. Three patients underwent inhibitory cathodic tDCS over the SSC and three had anodic excitatory tDCS over the MC, both contralateral to the side of the TN.

Functional MRI-based navigation was used to identify sites within the SSC and MC corresponding to the region of facial pain. The precise locations of tDCS electrodes were determined using a transcranial magnetic stimulation (TMS)-based navigator (LOCALITE GmbH, Bonn, Germany, Personal Communication). A high spatial resolution T1-MPRAGE MRI with voxel size 1 mm^3^, 3D brain segmentation and registration tools confirmed the stimulation target sites for subsequent imaging and therapeutic sessions. A conductive paste (Ten20) was manually applied as a conductor between the electrodes and skin, with the resultant impedance checked for the subject’s safety. MR-compatible tDCS equipment (NeuroConn DC-stimulator) was used to apply 2 mA direct current concurrently with fMRI paradigm. A pair of MR-compatible rectangular rubber electrodes with dimensions of 35 cm^2^ (7 × 5) were used with rubber band fixation to keep the electrodes in place during imaging. The pretreatment session with fMRI consisted of the standard three stages in the block-design imaging analysis approach: (1) “tDCS” stimulation task targeting optimal regions in the block design; (2) Pain stimulation task with pain triggered in the block design; (3) “Pain + tDCS” stimulation task with pain triggered instantaneously with tDCS in the block design between rest phases. During activation blocks in the “Pain” task, the patients were instructed to bite their ipsilateral interior cheek next to the second upper molar tooth in order to reproduce their neuralgiform pain in the territory of trigeminal nerve. The same scenario was applied for the “Pain + tDCS” task, while an electrical stimulus was directed to the motor (anodic/excitatory stimulation) or sensory (cathodic/inhibitory stimulation) cortex. For the “tDCS” task, only the electrical current was applied during activation blocks with the patients instructed to do nothing during the task. A diffusion MRI (dMRI) study was also performed in the pretreatment phase. The patients then underwent five sessions of tDCS treatment with either cathodic stimulation delivered onto the contralateral SSC or anodic stimulation upon the contralateral MC. Again, a direct current of 2mA was applied by the same tDCS for a 20-min duration with 30s fade in/out. A saturated sponge with normal saline (0.9%) was used as a conductor between the electrode and skin with impedance checking for the subject’s safety. A one-day interval was provided between sessions and the posttreatment study was performed two days after the last treatment session. Both fMRI and DTI were performed with the same tasks and imaging parameters as in the initial study. The Numeric Rating Scale (NRS) and the Headache Disability Index (HDI) were used to score the subjective efficacy of treatment. NRS and HDI were registered before and one week after completion of the study (Jacobson et al., 1994).

### Imaging Protocols, Preprocessing, and Analysis

All subjects underwent high-resolution structural MRI and fMRI block-design stimulation tasks, along with a single-shell dMRI acquisition using a 64-channel phased array head coil and a 3-Tesla scanner (Siemens Prisma, Erlangen, Germany) using the software version “Syngo MR E11.” A structural MRI was acquired using a standardized protocol as follows: transverse T1-weighted images using the MPRAGE protocol with imaging parameters, TR = 1840 ms, TI = 900 ms, TE = 2.43ms, flip angle = 8°, matrix = 224 × 224, in-plane resolution = 1.0 × 1.0 mm^2^, slice thickness = 1.0 mm, pixel bandwidth = 250 Hz/pixel. The volumes of the task-related fMRI (120 measurements) covering the whole brain were acquired in the transverse plane using an echo planar imaging (EPI) sequence (TR = 3,000 ms, TE = 30 ms, flip angle = 90, matrix = 640 × 640, slice thickness = 2.4 mm). In the “Pain” stimulation task, sic blocks of activation were applied, each followed by a rest period. The duration of each fMRI task block was 6 min. Single shell dMRI (b-value of 1,000 s/mm^2^ involved 64 diffusion gradient directions acquired using EPI with the same unit in an anteroposterior phase direction with the following imaging parameters: TR = 9,600 ms, TE = 92 ms, flip angle = 90, matrix = 110  110, in-plane resolution = 2.0 × 2.0 mm^2^, slice thickness = 2.0 mm, pixel bandwidth = 1,420 Hz/pixel. Two sets of null volumes using the above-mentioned imaging parameters and without applying diffusion synthesizing gradients (b-value of 0 s/mm^2^ were acquired using two opposite phase-encoding directions (AP and PA) to perform distortion correction.

Preprocessing steps included motion correction and eddy current correction, followed by a two-step registration protocol. First, each dMRI or fMRI volume was registered to its own T1 space. A transformation of dMRI or fMRI was then undertaken to register the individual subject’s T1 space to standard MNI space. After preprocessing, FEAT in FSL tools (Linux) was used to extract the activation areas for each of the three tasks. The ExploreDTI (v4.8.6) package in MATLAB 2015b was used to perform tensor fitting for all subjects and establish index maps for FA and MD.

To determine the effectiveness of tDCS upon network activation, particular sites such as the caudate, SSC, globus pallidus GP, putamen, thalamus, and cingulate gyrus, believed to be influenced by TN, were extracted using FEAT in FSL. Both MD and FA were determined at the trigeminal root entry zone (REZ). The number of sensory fibers, speculated to be involved in the pain propagation of TN, were extracted using ExploreDTI and manual ROI insertion. Finally, the method of constraint spherical deconvolution (CSD) was implemented for analysis of high angular resolution of dMRI data and fiber tracking (Leemans et al., 2009; Jeurissen et al., 2010, 2011; Kristo et al., 2013).

### Statistical Assessment: Effect of Pain, tDCS Stimulation and Treatment

We assessed the effect of unilateral pain application upon ipsilateral vs. contralateral brain structures with the “Pain” stimulation task alone. This was followed by assessing the sites of activation generated by application of the “Pain + tDCS” task expected to bring about a dynamic suppression of pain induced. The effect of tDCS treatment upon site activation was examined in pre- and posttreatment sessions.

## Results

### Assessment of Treatment Efficacy

**FIGURE 1:**
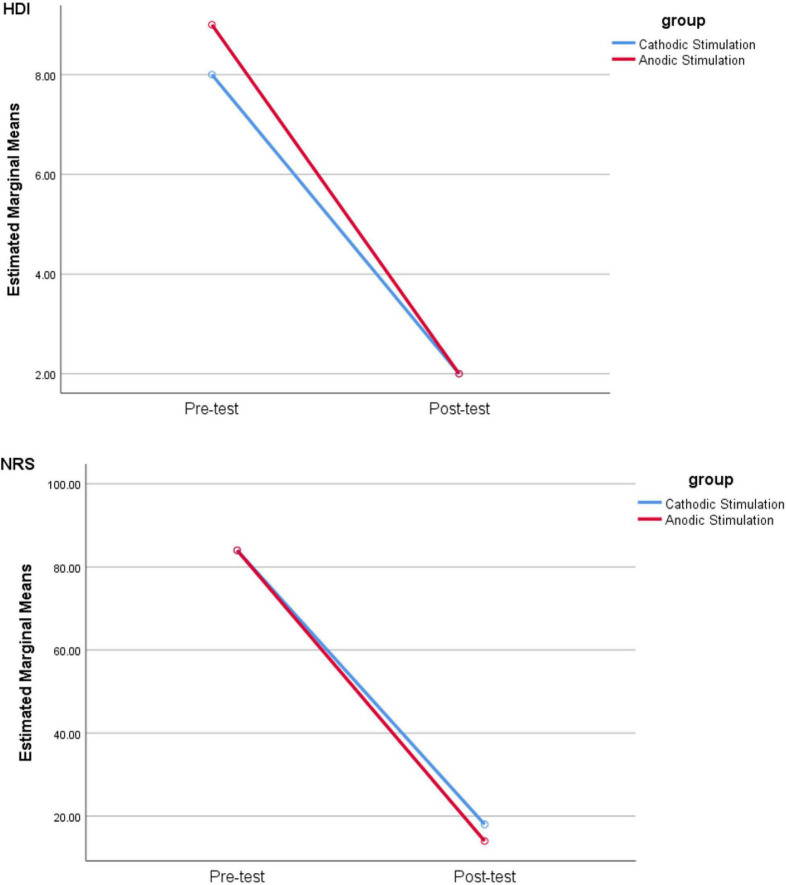
Comparison of HDI and NRS scores in pre- and postsessions by cathodic and anodic stimulation. Note that NRS is used to quantify the severity of pain stipulating “0” as no pain and “10” as the greatest imaginable pain, while HDI provides a score for the level of disability suffered with TN.

Repeated measurement analysis showed no differences between groups with cathodic and anodic stimulations for HDI (*F* test = 0.237, *P* = 0.6) and NPRS (*F* test = 0.14, *P* = 0.7). Conversely, significant differences for HDI (*F* test = 190.125, *P* < 0.001) and NRS (*F* test = 224.733, *P* < 0.001) (Figure 1 and Supplementary Table 2) were found between pre- and postintervention.

### Assessment of Unilateral Pain Upon Activation of Brain Structures

A significantly greater activation was found with anodic tDCS, after Bonferroni adjustment, of the caudate and SSC within the ipsilateral pain zone compared to that of the contralateral side as determined in the pretreatment session. No significant difference was identified, otherwise, in any other brain structure in the posttreatment session.

### Assessment of Pain Suppression Effects of tDCS Upon Brain Structures

Concurrent application of tDCS stimulation during the “Pain task” (“tDCS + Pain”) in the pretreatment session significantly reduced activation in both ipsilateral and contralateral caudate and ipsilateral SSC in cases of anodic tDCS treatment. The same occurred in both ipsilateral and contralateral thalamus in cases treated by cathodic tDCS treatment. There was no significant difference between “Pain” and “Pain + tDCS” stimulation tasks for these structures during the posttreatment session (Supplementary Figure 1 and Supplementary Table 2). The findings of fMRI correlated with the effectiveness of tDCS of both MC and SSC using anodic and cathodic stimulation, respectively.

### Transcranial DCS Application

A significant increase in activation was found within the caudate and putamen bilaterally with anodic stimulation. A similar significant increase in activation was identified in the contralateral caudate, GP, lateral SSC, and in the putamen bilaterally with cathodic stimulation (Supplementary Figure 1 and Supplementary Table 2).

### Assessment of Therapeutic Effects of tDCS

Following a course of five tDCS treatments, regardless of stimulation methodology, we observed a significant decrease in activation of the ipsilateral caudate, SSC, GP, and thalamus, and in the contralateral GP during the posttreatment session compared to that of pretreatment. With anodic stimulation, the observed significant decrease from pre- to posttreatment occurred only in the ipsilateral caudate and SSC. On the other hand, cathodic stimulation brought about increased activation in the ipsilateral thalamus. The decrease within the ipsilateral and contralateral GP occurred with both anodic and cathodic applications. These observations prompted investigation of anodic and cathodic cases separately. For cases of anodic stimulation, a significant decrease in activation occurred in the ipsilateral caudate, SSC and GP, and the contralateral GP. With cathodic stimulation, a significant decrease in activation was observed in the contralateral SSC, GP, thalamus, and anterior cingulate gyrus (ACG), and the ipsilateral GP and thalamus in the posttreatment compared to the pretreatment situation (Supplementary Figure 1 and Supplementary Table 4).

The dMRI analysis showed a substantial increase (>50%) in the number of contralateral sensory fibers in the spinothalamocortical sensory tract (STCT) following the complete course of tDCS stimulation in three of the six patients (Supplementary Figures 2A,B). A significant reduction in fractional anisotropy (FA) (>40%) was observed in the ipsilateral trigeminal REZ, after Bonferroni adjustment, in the posttreatment phase compared to pretreatment in five of the six patients (Supplementary Figure 3). Three patients showed a substantial increase (>32%) while a single case was found to have a relatively minor decrease (13%) in FA within the contralateral REZ in the posttreatment phase (Supplementary Figure 3). A reduction in mean diffusivity (MD) (>12%) within the contralateral REZ in the posttreatment phase was a consistent finding across all patients (Supplementary Figure 4).

## Discussion

The concurrent application of anodic tDCS and a relevant pain stimulus significantly attenuates activation in both the ipsilateral and contralateral caudate and the ipsilateral SSC. Likewise, following a course of anodic tDCS, the ipsilateral caudate, GP and SSC and the contralateral GP showed a significantly attenuated activation as assessed by the “Pain” stimulation task. Stimulation rendered within the TN pain zone revealed an area of activation within the ipsilateral caudate and SSC that was significantly greater when compared to the contralateral side. This difference disappeared following a course of anodic tDCS treatment. Concurrent application of cathodic tDCS and pain stimulation significantly reduced activation in both ipsilateral and contralateral thalamus. After a course of cathodic tDCS treatment, a significant decrease with the “Pain” stimulation task was identified in the thalamus and GP bilaterally, and the SSC and ACG contralaterally.

Findings in the current study are consistent with prior formulations of a pain neuromatrix (Moissetl et al., 2011). The remarkable feature here, however, was the significant success achieved with both cathodic and anodic tDCS in relieving pain. The functional outcomes achieved with activation of different sets of anatomical elements by each of the two neuromodulatory applications implicate different mechanisms in achieving the same effect.

### FMRI Findings: Cathodic tDCS Stimulation

**FIGURE 2:**
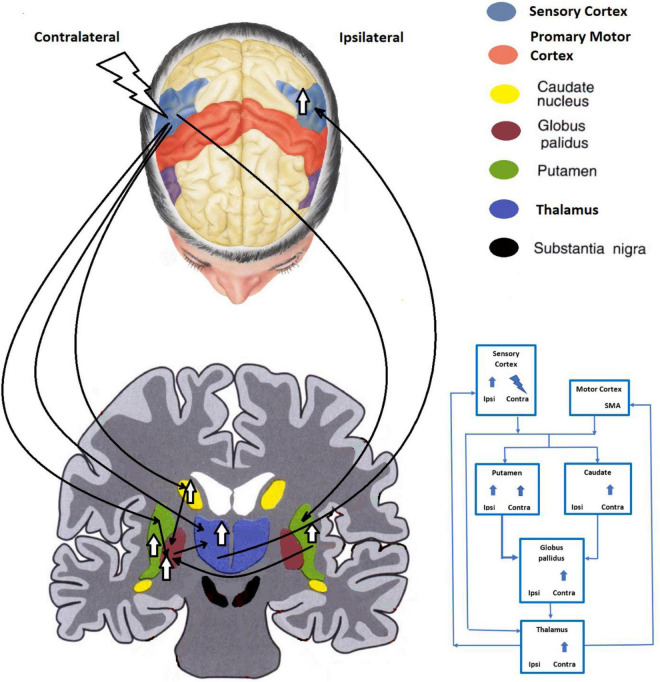
The proposed cathodic tDCS modulation pathway of the contralateral sensory area.

Cathodic tDCS of the SSC alters activity within the ipsilateral SSC, the contralateral caudate, GP and thalamus and the putamen bilaterally (Figure 2). A proposed pathway makes use of the GP-thalamus-cerebral cortex circuit previously described (Alexander et al., 1986). Unilateral cutaneous stimulation activates the SSC bilaterally^35^, although the effect is more contralateral. Application of tDCS tends to equalize the activation. After a course of treatment, the pain score during pain induction is reduced significantly, along with a reduced activation of the GP and thalamus bilaterally, and the contralateral SSC and ACG (Rainville et al., 1997; Yesudas and Lee, 2015).

The cathodic tDCS course of treatment resulted in greater contralateral thalamic activation compared to pretreatment acquisition. With pain induction, the thalamus bilaterally was shown to manifest less activation. Such relative hypoactivity would be interpreted subjectively as a reduced sense of pain. The final outcome could be attributable to a conveyance of lesser input to the sensorimotor cortex. Transcranial DCS alone increased bilateral putamenal activation in both phases, more significantly on the ipsilateral side. The subjective perception of pain has been shown to be influenced by putamenal activation with a functional connection identified between it and the sensorimotor area (Starr et al., 2011). An animal model has also shown activation of striatal dopamine receptors to be effective in suppressing induced pain (Lin et al., 1981; Magnusson and Fisher, 2000). In this regard, patients with Parkinson’s disease have been presumed to have greater susceptibility toward pain because of the progressive loss of nigrostriatal dopaminergic neurons (Defazio et al., 2008; Cheon et al., 2009). Altogether, these studies support the important role of striatal dopamine in processing pain. Hence, cathodic tDCS of the contralateral SSC may exert its mitigation of pain through the activation of the putamen.

### fMRI Findings: Anodic tDCS Stimulation

**FIGURE 3:**
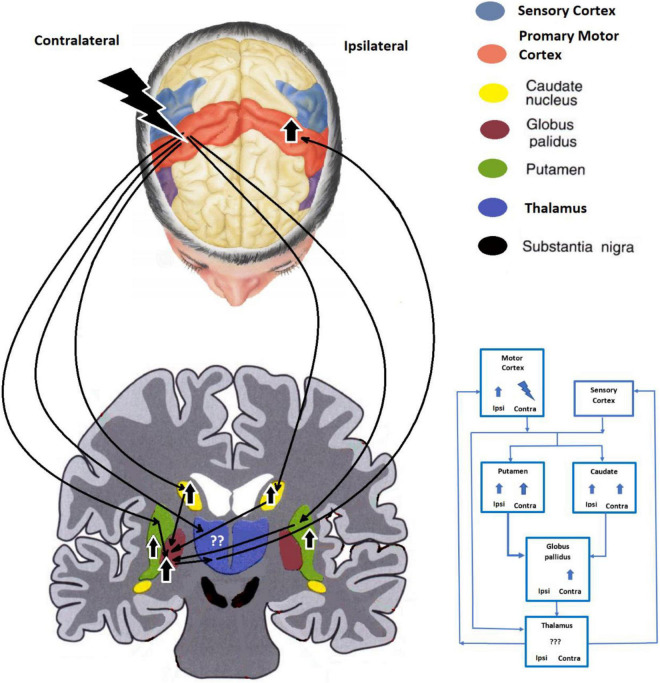
The proposed anodic tDCS modulation pathway of the contralateral motor area.

In cases undergoing anodic tDCS stimulation of the contralateral MC, activation of the caudate and putamen bilaterally and the contralateral GP ostensibly brought about a pain suppressive effect. The net pain-relieving effect was reflected in the ipsilateral SSC and caudate and GP bilaterally in the posttreatment phase (Figure 3).

Prior to application of the treatment protocol, anodic stimulation of the MC with simultaneous pain induction showed both caudate nuclei to be activated much less than by pain induction alone. Following treatment, pain delivery resulted in less activation of the caudate ipsilaterally than during the pretreatment phase along with a corresponding subjective relief of pain. Suppression of the caudate and the resultant suppression of the thalamus again would bring about the same pain mitigation as with cathodic tDCS stimulation.

In a previous study, painful electrical stimulation accompanied by a suppression task in the early phase of stimulation (i.e., initial 13 s) resulted in bilateral caudate activation while in the late phase (i.e., after 39 s) caudate activation was reduced significantly (Wunderlich et al., 2011). The authors interpreted the activation in the early phase as a feature of the pain-suppression task or an effort required to suppress the pain. Another study using painful thermal stimulation accompanied by a suppression task resulted in bilateral caudate activation in the early phase of pain suppression while activation of the prefrontal cortex was identified in the late phase. Here, the caudate was implicated in the initiation of pain suppression and the prefrontal cortex in its maintenance (Freund et al., 2009).

Caudate activation by tDCS is consistent with these findings. The subsequent reduced activation of the caudate after anodic tDCS treatment coincides with the sustained subjective relief of pain in our cases. Caudate suppression and its putative disinhibition of the thalamus (Chevalier and Deniau, 1990) would ultimately bring about a reduction of SSC activity. Putamenal activation is known to have a similar effect (Lin et al., 1981; Magnusson and Fisher, 2000; Starr et al., 2011). Likewise, activation of the GP in the same manner would influence thalamocortical activity resulting in the same outcome.

### dMRI Findings

Reduced FA within the ipsilateral and reduced MD within the contralateral trigeminal root entry zone following a course of tDCS in the majority of patients suggests an induced neuroplasticity coinciding with pain relief. This partly supports but also contrasts with findings of a previous study that investigated structural changes in TN after decompression surgery which showed reduction in FA and an increase in MD at the affected trigeminal REZ (Zhang et al., 2018) and may point to differences in effect brought about by cerebrocortical neuromodulation (Jürgens, 1984). The quantity of fibers within the STCT was also substantially increased indicating a more widespread effect. In another study, episodic central pain was evaluated in a patient with multiple sclerosis and demonstrated an abnormal unilateral temporary FA increase in the thalamus contralateral to the affected body side and normal values pre- and postepisodic pain (Attal et al., 2016). This finding implies the increase of directional uniformity of the water diffusion in the thalamus, perhaps due to the participation and integration of more fiber tracts during the pain which is comparable with our findings in the ipsilateral trigeminal nerve pre- and post-tDCS intervention. The increased sensory fiber number in the STCT does raise support for the notion that cerebrocortical reinnervation may play a significant role in mitigating the symptoms of TN.

### Limitations

Limitations of this study include the absence of a control group and determination of a placebo effect related to the application of tDCS. Although the imaging features accompanying the positive findings in the study remain compelling, the sample size is insufficient to declare efficacy and further study of the effect with a larger patient population is required. A fatigue aspect in the study may have affected a number of patients and contributed to a desire for completion of the study prematurely resulting in a bias toward a favorable effect. In addition to a larger sample size of randomized patients treated with cathodal and anodal tDCS (e.g., *N* = 12), a separate cohort undergoing sham treatment is advisable to evaluate any placebo effects.

Patients in the current study were not subject to any recent changes in medical therapy and served as their own control. Nevertheless, future study must also exclude any mitigation of the therapeutic effect by psychological comorbidity or the effect of any medical treatment.

## Data Availability Statement

The raw data supporting the conclusions of this article will be made available by the authors, without undue reservation.

## Ethics Statement

The studies involving human participants were reviewed and approved by Tehran University of Medical Sciences, Ethical Board. The patients/participants provided their written informed consent to participate in this study.

## Author Contributions

BB contributed in proposing the research idea, providing the research funding, and writing the manuscript’s Introduction. NT contributed in designing and carrying out the research, supervising the data processing, neuroscience modeling, and writing the manuscript’s Discussion. KE contributed in data interpretation and revising and finalizing the manuscript. NS contributed in carrying out the fMRI task and tDCS treatments on the patients in therapeutic sessions. MB contributed in carrying out the MRI data acquisition. FE and NM contributed in functional MRI data analysis. ZK and AT contributed in proposing the research idea and patient recruitment. M-RN-Z contributed in conducting the research, supervising the data acquisition and processing, and writing the manuscript’s Summary, Methods, and Results. All authors contributed to the article and approved the submitted version.

## Conflict of Interest

MB was employed by Intelligent Quantitative Biomedical Imaging L.L.C. The remaining authors declare that the research was conducted in the absence of any commercial or financial relationships that could be construed as a potential conflict of interest.

## Publisher’s Note

All claims expressed in this article are solely those of the authors and do not necessarily represent those of their affiliated organizations, or those of the publisher, the editors and the reviewers. Any product that may be evaluated in this article, or claim that may be made by its manufacturer, is not guaranteed or endorsed by the publisher.
