# Supplementary material for: A Preliminary Study of the Efficacy of Transcranial Direct Current Stimulation in Trigeminal Neuralgia
Source: Front Hum Neurosci. 2022 Mar 4;16:848347. doi: 10.3389/fnhum.2022.848347 (PMC8931809; doi:10.3389/fnhum.2022.848347)
Supplement: Supplementary file 1 [file Data_Sheet_1.PDF]

Supplementary Materials

Table S1. Demographic characteristics of the patients

TN cases	Age (years)	sex	Pain side	NRS	Type of the stimulation	Chronicity (years)
**Case 1**	64	female	left	9	Anodic	8
**Case2**	81	male	right	10	Anodic	10
**Case3**	38	male	left	7	Anodic	3
**Case4**	38	male	left	9	Cathodic	4
**Case5**	63	female	left	8	Cathodic	4
**Case6**	73	female	left	7	Cathodic	7

NRS: Numeric Rating Scale of pain during episodes of neuralgiform episodes.

Table S2. HDI and NRS scores for differentiating TN and normal subjects

	group	N	Pre	Post	P value Within-Subjects	P value between-Subjects
**HDI (%)**	**Cathodic Stimulation**	3	8.7±1.5	2.0±2.0	0.002	0.6
	**Anodic Stimulation**	3	9.0±1.0	2.7±0.58	0.02	
**NRS**	**Cathodic Stimulation**	3	84.7±7.02	18.0±7.2	0.01	0.7
	**Anodic Stimulation**	3	84.7±10.07	14.7±6.4	0.004	

HDI= Headache Disability Index

NRS= Numeric Rating Scale

Table S3. Assessment of pain suppression effects of tDCS stimulation in the brain structures.

Structures	Sides	Pain vs Pain + tDCS in All Cases		Pain vs Pain + tDCS in Cases Treated by Anodic tDCS		Pain vs Pain + tDCS in Cases Treated by Cathodic tDCS	
		**Pretreatment**	**Posttreatment**	**Pretreatment**	**Posttreatment**	**Pretreatment**	**Posttreatment**
**Caudate**	**Ipsi**	P > PT*	-	P > PT**	-	-	-
	**Contra**	P > PT*	-	P > PT**	-	-	-
**SSC**	**Ipsi**	-	-	P > PT**	-	-	-
	**Contra**	-	-	-	-	-	-
**Thalamus**	**Ipsi**	P > PT*	-	-	-	P > PT**	-
	**Contra**	P > PT*	-	-	-	P > PT**	-

The table shows the significant difference in activated areas in “Pain” vs “Pain + tDCS” stimulation tasks in both pre- and posttreatment sessions. *Significant before Bonferroni adjustment. **Significant after Bonferroni adjustment.

Structures of interest: caudate, somatosensory cortex (SSC), globus pallidus (GP), putamen, thalamus and cingulate gyrus (CG).

Table S4. Assessment of therapeutic effects of tDCS

Structures	Sides	Degree of Pain in All Cases	Degree of Pain Treated by Anodic tDCS	Degree of Pain Treated by Cathodic tDCS	tDCS in All Cases	tDCS in Cases Treated by Anodic tDCS	tDCS in Cases Treated by Cathodic tDCS
**Caudate**	**Ipsi**	Pre > Post *	Pre > Post **		Pre < Post **	Pre < Post **	
	**Contra**				Pre < Post **	Pre < Post **	Pre < Post **
**SSC**	**Ipsi**	Pre > Post *	Pre > Post **		Pre < Post **		Pre < Post **
	**Contra**			Pre > Post **			
**GP**	**Ipsi**	Pre > Post **	Pre > Post **	Pre > Post *			
	**Contra**	Pre > Post *	Pre > Post *	Pre > Post **	Pre < Post **	Pre < Post **	Pre < Post **
**Putamen**	**Ipsi**				Pre < Post **	Pre < Post **	Pre < Post **
	**Contra**				Pre < Post **	Pre < Post **	Pre < Post **
**Thalamus**	**Ipsi**	Pre > Post *		Pre > Post **			
	**Contra**			Pre > Post *	Pre < Post **		Pre < Post **
**CG**	**Ipsi**						
	**Contra**			Pre > Post *			

The table shows differences in activated areas in pre- vs posttreatment sessions by the “pain” stimulation task. *Significant before Bonferroni adjustment. **Significant after Bonferroni adjustment.

Structures of interest: caudate, somatosensory cortex (SSC), globus pallidus (GP), putamen, thalamus and cingulate gyrus (CG).

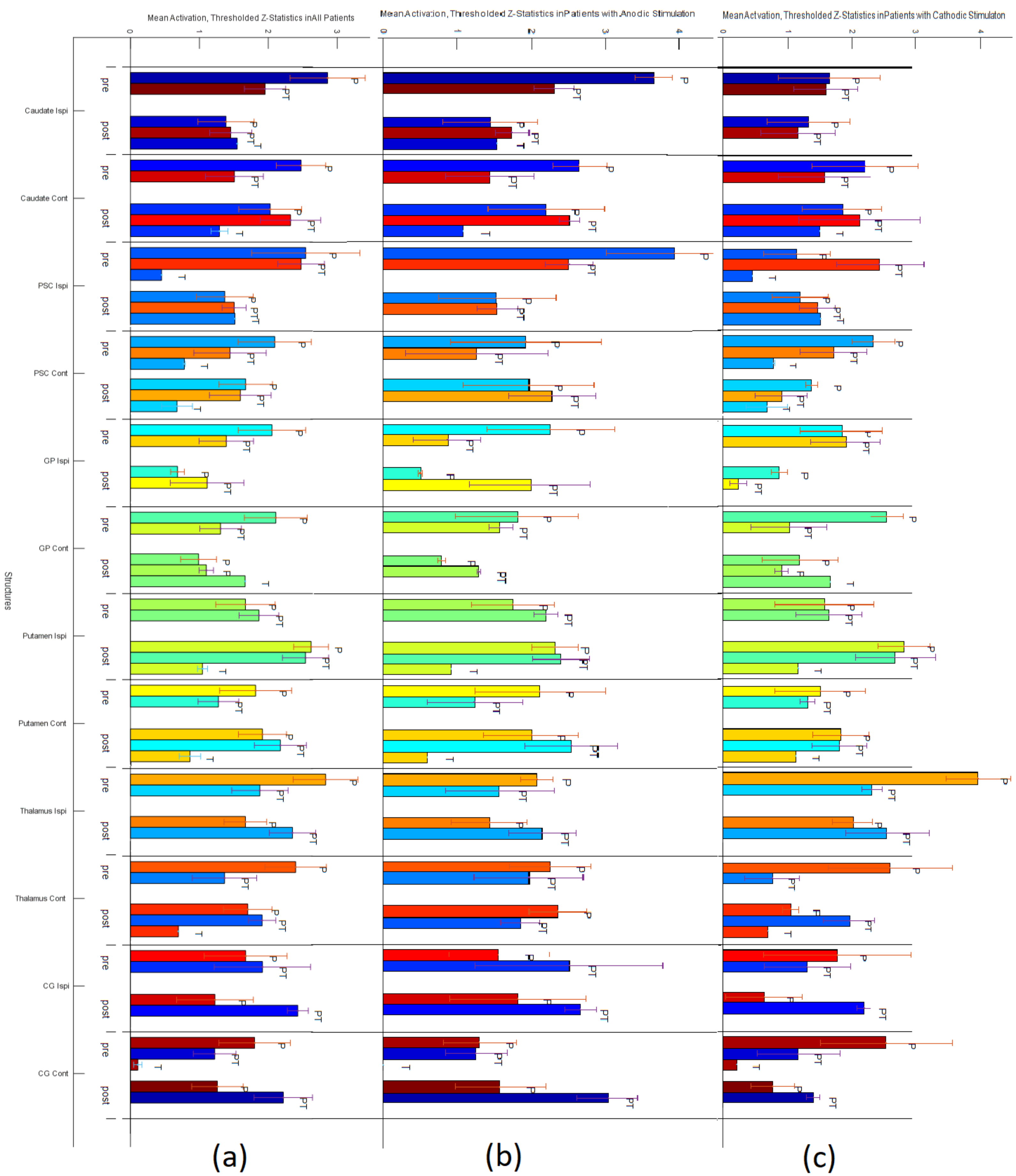
Mean Activation, Thresholded Z-Statistics in All Patients
Mean Activation, Thresholded Z-Statistics in Patients with Anodic Stimulation
Mean Actvation, Thresholded Z-Statistics in Patients with Cathodic Stimulaton
Structures
Caudate Ispi
Caudate Cont
PSC Ispi
PSC Cont
GP Ispi
GP Cont
Putamen Ispi
Putamen Cont
Thalamus Ispi
Thalamus Cont
CG Ispi
CG Cont
pre
post
P
PT
T
(a)
(b)
(c)

Fig. S1. Extent of fiber loss and network manifestations of tDCS: caudate, somatosensory cortex (SSC), globus pallidus (GP), putamen, thalamus and cingulate gyrus (CG). Comparison of Z-thresholded pain activations can be made for ipsi (ipsilateral) vs contra (contralateral) brain structures, P ("Pain") vs PT ("Pain + tDCS"), and pre (pretreatment) vs post (posttreatment) for (a): all patients; (b): patients treated by anodic tDCS; and (c): patients treated by cathodic tDCS. The colored bars represent mean and the error bars, standard deviation of activation across all patients.

Patients #	TN Side<br>Increment (%)	Pre<br>R L	Post<br>R L
1	Left<br>Right (72%)	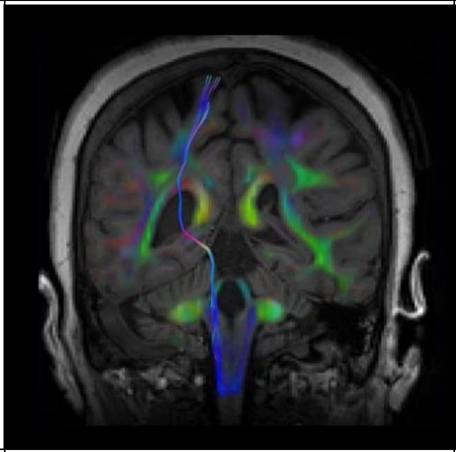	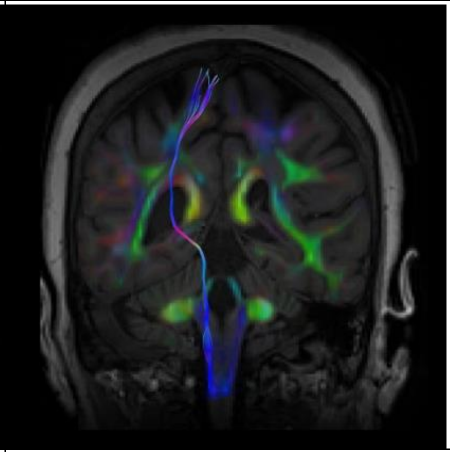
2	Right<br>Left (51%)	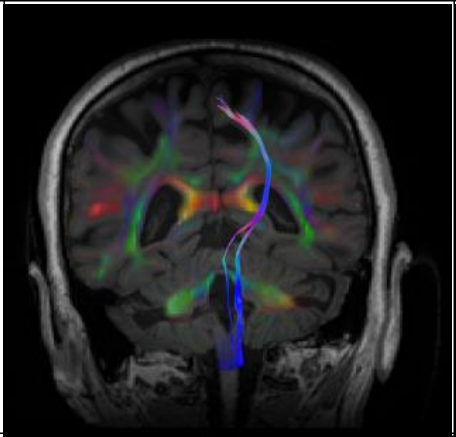	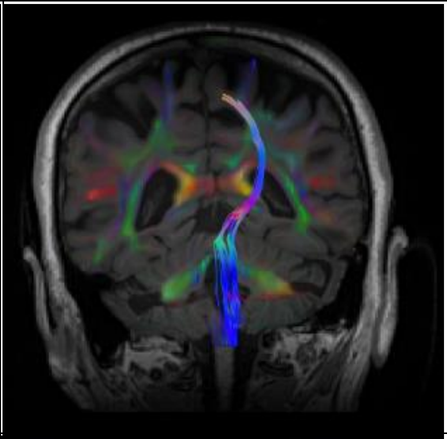
3	Left<br>Right (16%)	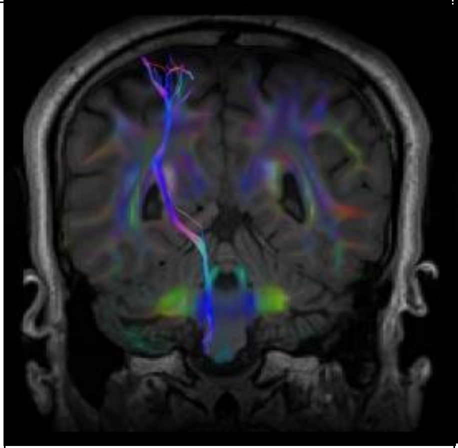	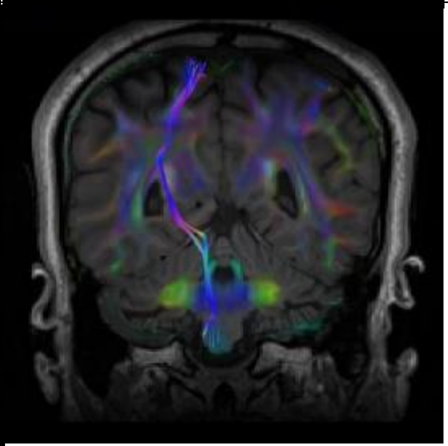

Patients #	TN Side<br>Increment (%)	Pre<br>R L	Post<br>R L
4	Left<br>Right (12%)	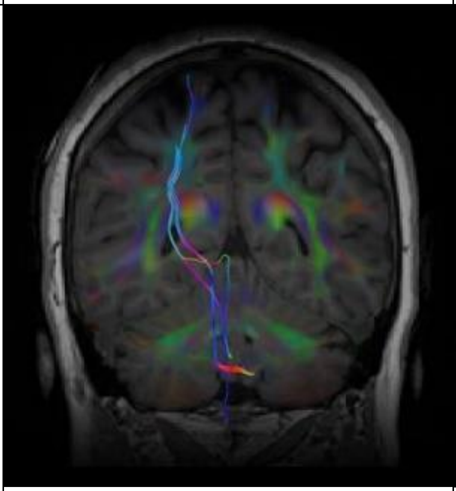	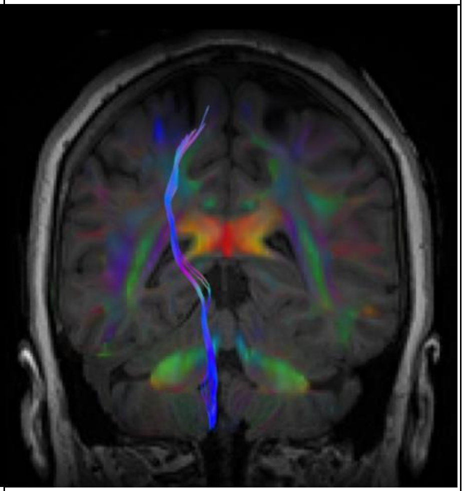
5	Left<br>Right (83%)	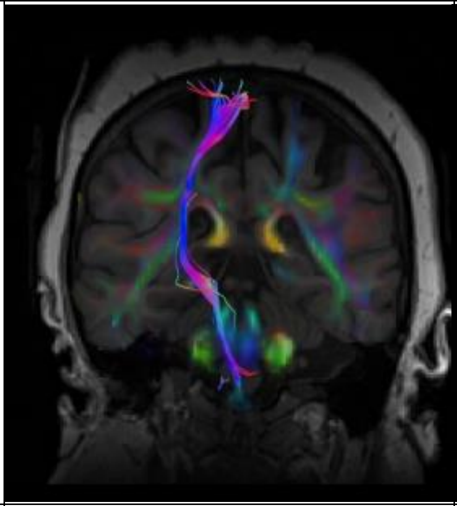	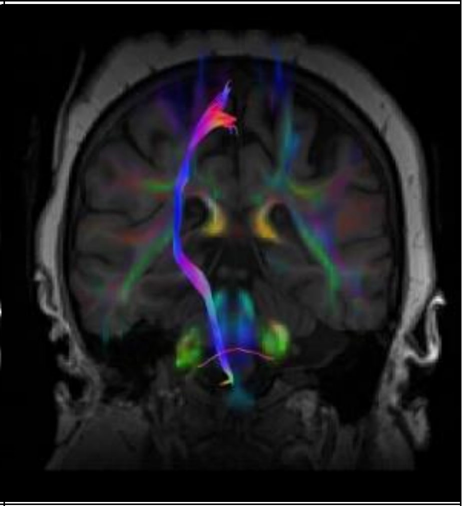
6	Left<br>Right (18X)	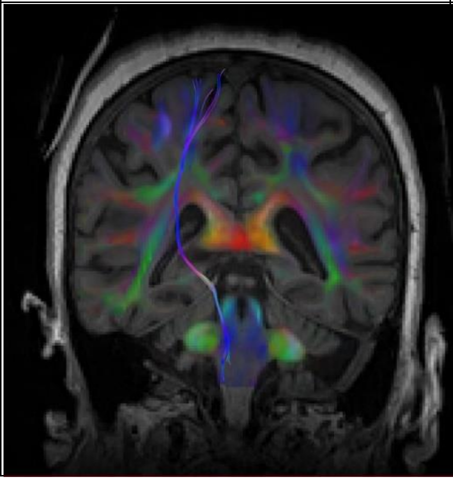	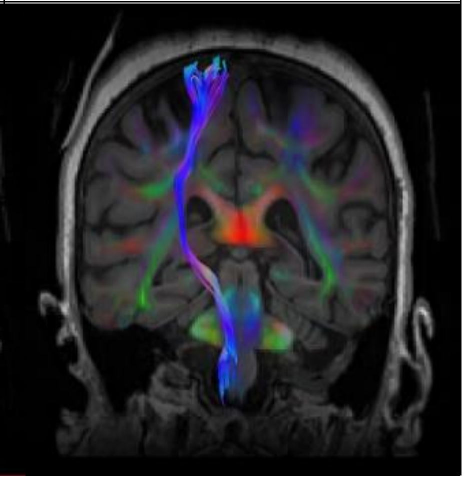

(A)

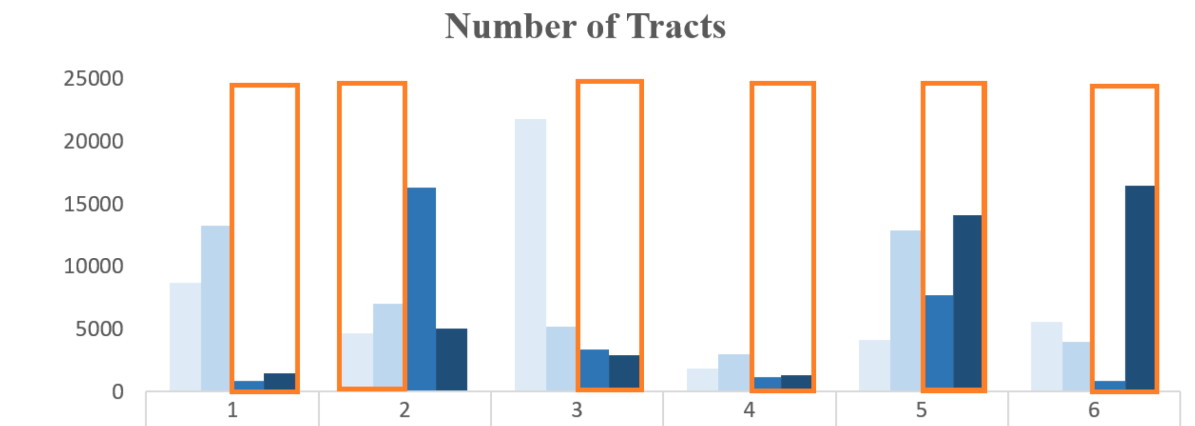

	1	2	3	4	5	6
pre_Left	8727	4646	21856	1817	4147	5616
post_Left	13263	7029	5239	2973	12903	3984
pre_Right	857	16358	3379	1184	7708	853
post_Right	1471	5054	2896	1335	14122	16449

(B)

Fig. S2. (A). Spinothalamocortical tractography identifying increases in the number of contralateral sensory fibers, with substantial changes noted in 4 of the 6 patients (cases 1, 2, 5, 6) following tDCS stimulation (i.e., posttreatment). (**B).** Bar graph illustrating the quantitative significance of these changes (orange rectangles in contralateral measures). Note that for all patients the side of PN was left, except for the patient #2.

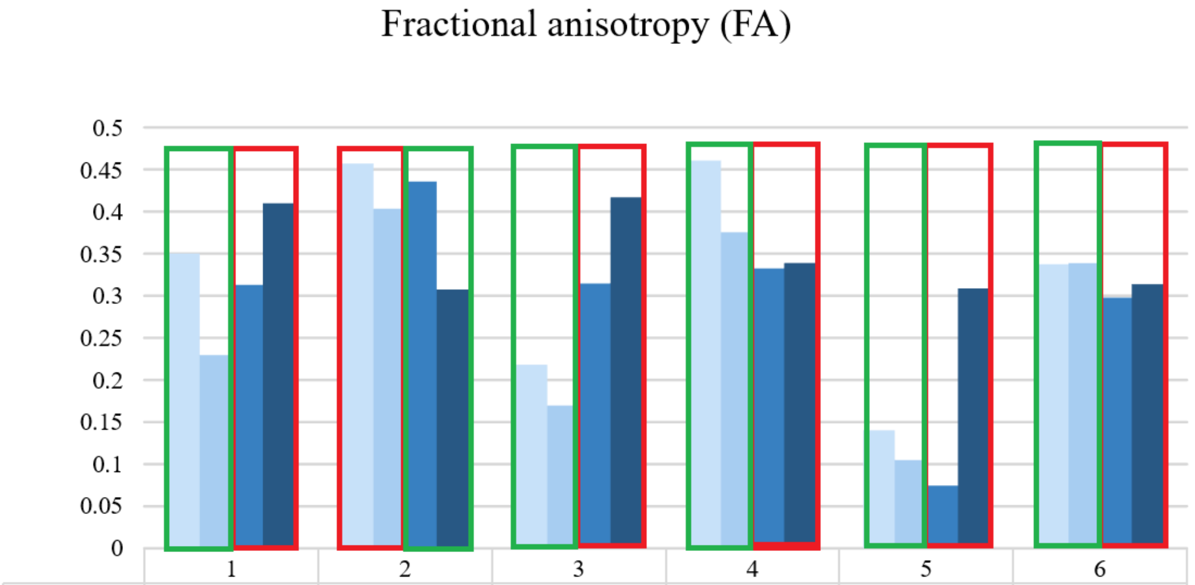

	1	2	3	4	5	6
pre_Left	0.35025	0.4569	0.21808	0.46049	0.14019	0.3375
post_Left	0.22955	0.40332	0.16969	0.37533	0.10486	0.33868
pre_Right	0.31288	0.43559	0.31447	0.33252	0.074432	0.29764
post_Right	0.40986	0.30743	0.4169	0.33894	0.30877	0.31372

Fig. S3. FA in the trigeminal root entry zone (REZ). Significant FA reductions were seen in the ipsilateral REZ (green rectangles) in the posttreatment phase in 5 of 6 patients after Bonferroni adjustment. A significant increase of FA was seen in the contralateral REZ (red rectangles) of 4 patients in the posttreatment phase while one showed a significant decrease.

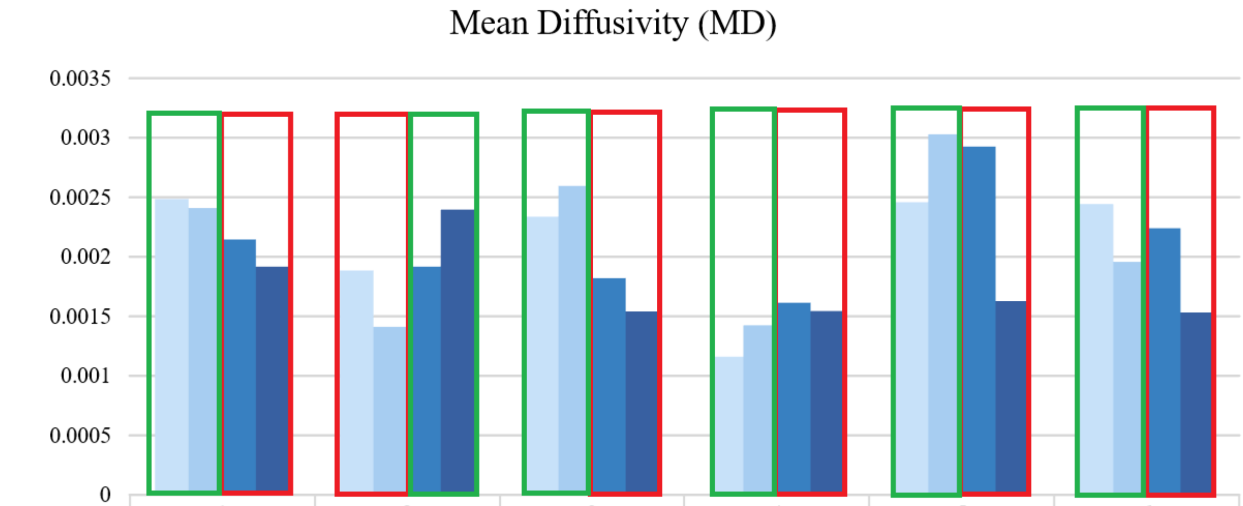

	1	2	3	4	5	6
pre_Left	0.0024849	0.0018841	0.0023358	0.0011597	0.0024582	0.0024431
post_Left	0.0024095	0.001411	0.0025948	0.0014246	0.003029	0.0019575
pre_Right	0.0021455	0.0019169	0.00182	0.0016134	0.0029255	0.0022395
post_Right	0.0019169	0.0023959	0.0015403	0.001544	0.0016271	0.0015316

Fig. S4. Mean diffusivity (MD) in the trigeminal root entry zone (REZ). A significant reduction in MD of the contralateral REZ (red rectangles) in the posttreatment phase was a consistent finding across all patients.
